# Proteinase 3 Antibody and Anti-Double-Stranded DNA in a Patient With Immunoglobin Light Chain Amyloidosis

**DOI:** 10.7759/cureus.47667

**Published:** 2023-10-25

**Authors:** Jessica K Cobb, Lachlan Shiver, Charles R Russell, Bo Chen, Claude Bassil

**Affiliations:** 1 Nephrology, University of South Florida Morsani College of Medicine, Tampa, USA; 2 Nephrology, University of South Florida (USF) Health Morsani College of Medicine, Tampa, USA; 3 Onco-Nephrology, H. Lee Moffitt Cancer Center, Tampa, USA; 4 Department of Pathology and Cell Biology, USF Morsani College of Medicine, Tampa, USA; 5 Nephrology and Hypertension, University of South Florida, Tampa, USA

**Keywords:** antineutrophil cytoplasmic antibody (anca), mpo-anca, al amyloidosis, dsdna, pr3

## Abstract

Proteinase 3 (PR3) anti-neutrophil cytoplasmic antibodies (ANCA) and anti-double-stranded DNA (anti-dsDNA) antibodies have been associated with a variety of nephritic diseases, most recognizably granulomatosis with polyangiitis and systemic lupus erythematosus (SLE) glomerulonephritis, respectively. We report the first clinical case of positive PR3 and dsDNA in a patient with renal Immunoglobin light chain (AL) amyloidosis. A 75-year-old man presented to the hospital with chronic fatigue, weight loss, and a recent diagnosis of left ventricular infiltrative cardiomyopathy secondary to AL amyloidosis. Autoimmune serology was significant for PR3-ANCA and anti-dsDNA antibodies. A renal biopsy confirmed AL amyloidosis with diffuse Congo red stain. This case report is the first of its kind, showing atypical antibody presentation in the setting of amyloidosis.

## Introduction

Amyloidosis is a disease in which normal serum protein subunits build up in organs. These proteins are often in anti-parallel beta-pleated sheets, which allow the protein deposition to be identified on electron microscopy; this yields green birefringence under polarized light when stained with Congo red. AL amyloidosis is the most common subtype of amyloidosis but is still rare, with a yearly incidence rate of nine cases per million persons [1]. AL amyloidosis protein subunits are comprised of fragments of monoclonal light chains. While AL amyloidosis may be systemic, one organ is often chiefly affected, most commonly the kidneys (74%) [2]. Diagnosis requires biopsy-proven amyloid fibril detection, commonly achieved with Congo red stain revealing green birefringence under polarized light [3]. To our knowledge, no publications of an AL amyloidosis patient with anti-dsDNA antibody or proteinase 3 anti-neutrophil cytoplasmic antibody (PR3-ANCA) involvement have been published. One published case report shows myeloperoxidase (MPO) ANCA-associated crescentic glomerulonephritis in an AL amyloidosis patient [4]. We present a case of biopsy-proven AL amyloidosis in a chronic kidney disease (CKD) stage 3b/A3 patient who was subsequently found to have positive PR3-ANCA and anti-dsDNA antibodies.

## Case presentation

A 75-year-old man with a past medical history significant for multiple myeloma, early Alzheimer's, hypertension, hypercholesterolemia, arthritis, CKD stage 3b/A3, and recurrent nephrolithiasis with calcium-based kidney stones (treated with lithotripsies) presented to our clinic to establish care for progressive loss of renal function. The patient was recently diagnosed with AL amyloidosis infiltrative cardiomyopathy with an estimated left ventricular ejection fraction (LVEF) of 47%.

On initial presentation, the patient complained of chronic fatigue and weight loss. Seven months prior, he presented to an outside hospital with COVID-19 complicated by acute renal failure and nephrotic range proteinuria. Labs at this time showed blood urea nitrogen (BUN) of 35 mg/dL, creatinine of 1.5 mg/dL, estimated glomerular filtration rate of 46 mL/min/1.73m^2^, urine protein random of 114.9 mg/dL, kappa quant free light chains of 64.40 mg/L, and lambda quant free light chains of 381.95 mg/L (Table 1). Workup at that time showed high serum lambda levels, and an aspirated bone marrow biopsy revealed mild marrow cellularity (50%) with trilineage hematopoiesis and mild atypical megakaryocytic. Twenty percent of cells stained positive for CD138, a marker consistent with plasma cells. Via in situ hybridization, it was determined that these cells produced lambda light chains. Flow cytometry performed on aspirate revealed monoclonal lambda-restricted plasma cells that stained positive for CD56 and CD117 and negative for CD19. Of note, amyloid was not present.

**Table 1:** The patient’s lab findings on the initial hospital encounter BUN = blood urea nitrogen; eGFR = estimated glomerular filtration rate; WBC = white blood cells.

Variable	Value	Ref Range & Units
Sodium	141	135 – 148 mEq/L
Potassium	4.5	3.5 – 5.3 mEq/L
Total CO2	29	22 – 29 mEq/L
BUN	35	6 – 20 mEq/L
Creatinine	1.5	0.72 – 1.25 mg/dL
eGFR	46	mL/min/1.73m^2^
Total protein	6.0	6.4 – 8.3 gm/dL
Albumin	3.6	3.5 – 5.0 gm/dL
WBC	7.89	4.6 – 10.2 10*3/uL
Kappa quant free light chains	64.40 mg/L	3.3 - 19.4 mg/L
Lambda quant free light chains	381.95 mg/L	5.7 - 2.63 mg/L

An endomyocardial biopsy performed shortly after showed focal amyloid protein deposition, focal interstitial fibrosis with minimal lymphocytic infiltration, and Congo red positivity without giant cell changes or viral inclusions. Mass spectrometry performed on the biopsy sample revealed AL lambda-type amyloid deposition. PET scan at this time demonstrated increased fluorodeoxyglucose uptake in the distal clavicle, which could be consistent with his multiple myeloma or osteolysis. The patient continued to follow up with cardiology. This concluded the patient’s initial admission.

Two months later, the patient was directly admitted for renal biopsy. On admission, the patient had continued complaints of chronic fatigue, weight loss, and dyspnea on exertion. The patient was hemodynamically stable, and his physical exam was entirely benign. Significant admission labs included a creatinine of 1.7 mg/dL (at baseline) and BUN of 38 mg/dL (Table 2). Urinalysis was negative for glucose, bilirubin, ketones, urobilinogen, nitrites, and leukocyte esterase with a specific gravity of 1.017, pH 5.0, 5-10 red blood cells (RBC) per high-powered field, and 100 mg/dL of protein (Table 3). Urine creatinine was 107.3 mg/dL with a protein/creatinine ratio of 1.27 and a microalbumin/creatinine ratio of 699 mcg/mg. Kappa and lambda quant free light chains were elevated at 61.34 mg/L and 449 mg/L, respectively, with a ratio of 0.14. Complement levels were within normal limits. Due to generalized constitutional symptoms coupled with arthritis, systemic symptoms, subnephrotic range proteinuria, and RBCs on urinalysis, we felt prudent to include nephritic disease and vasculitis on the differential. PR3-ANCA reflex was high at 29 U/mL, and MPO-ANCA was normal. dsDNA antibody was evaluated with two separate tests: enzyme-linked immunosorbent assay 39 U/mL and Crithidia luciliae indirect fluorescent test <1:10. ANA was negative (Table 2).

**Table 2:** The patient's lab findings on direct admission BUN = blood urea nitrogen; MCV = mean corpuscular volume; BNP = B-type natriuretic peptide; ANA = antinuclear antibody; MPO = myeloperoxidase; PR3 = proteinase 3; ANCA = anti-neutrophilic cytoplasmic antibody.

Variable	Value	Ref Range & Units
Creatinine	1.7 (at baseline)	0.72 – 1.25 mg/dL
BUN	38	6 – 20 mEq/L
Total protein	5.5	6.4 – 8.3 gm/dL
Albumin	2.8	3.5 – 5.0 gm/dL
Hemoglobin	11.1	14.1 - 18.1 g/dL
Hematocrit	34.7	43.5 - 53.7 %
MCV	96.7	80 - 97 fL
BNP	511.5	<100 pg/mL
Kappa quant free light chain	61.34	3.3 - 19.4 mg/L
Lambda quant free light chain	449	5.7 - 2.63 mg/L
Kappa/Lambda ratio	0.14	0.26 – 1.65
PR3-ANCA reflex	28	< 1.0 AI
MPO-ANCA	0	< 1.0 AI
dsDNA ELISA	<1:10, 39 U/mL	<30 IU/mL
ANA	None detected	Negative
Serum protein electrophoresis albumin	3.29	3.2 – 5.0 gm/dL

**Table 3:** The patient's urine studies on admission RBC = red blood cells.

Variable	Value	Ref Range & Units
Glucose	Negative	Negative mg/dL
Bilirubin	Negative	Negative mg/dL
Ketones	Negative	Negative mg/dL
RBC	5-10	0 – 2/HPF
Urobilinogen	Negative	<2 mg/dL
Nitrites	Negative	Negative
Leukocyte esterase	Negative	Negative
Specific gravity	1.017	1.007 - 1.030
pH	5.0	mg/dL
Protein	100	Negative mg/dL
Creatinine	107.3	mg/dL
Urine protein/creatinine ratio	1.27	<0.2
Microalbumin/creatinine ratio	699	<30 mcg/mg Cr
Urine protein random	114.9	0 – 14 mg/dL

**Figure 1:**
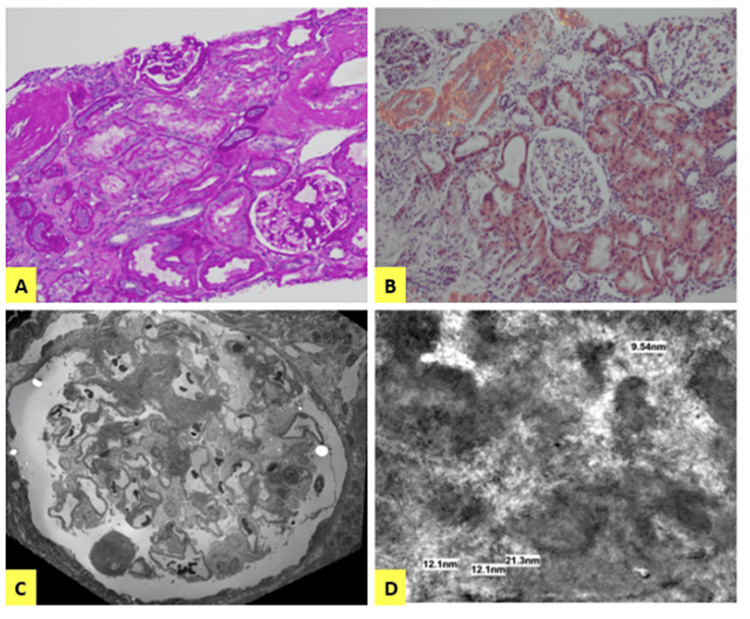
Histopathologic characters of renal amyloidosis (A) Periodic acid-shift (PAS) stain showing amorphous and extracellular material mainly involving blood vessel walls, focal interstitium, and occasional glomerular mesangium. (B) Characteristic apple-green birefringence under polarization with Congo red stain. (C) Low power electron microscopy (EM) showing mesangial deposition of randomly arranged, non-branching fibrils. (D) High-power electron microscopy (EM) showing mesangial deposition of randomly arranged, non-branching fibrils with a mean diameter of 12.26nm; characteristic of amyloid protein.

Congo red staining of renal biopsy confirmed AL amyloidosis that diffusely involved the vasculature and interstitium (Figures 1A-1D). Glomeruli was also involved, albeit to a lesser extent. Mild atherosclerosis, moderate to severe arteriolar hyalinosis, and mild tubular atrophy were also noted. A small subset of the intact glomeruli showed segmental, mild nodular mesangial expansion with weakly PAS-positive, silver-negative, eosinophilic acellular amorphous material. Acellular materials were also noted to replace portions of the interstitium focally, and the remaining tubulointerstitium showed mild acute tubular injury (Figure 1). There were no crystals, casts, or syncytial giant cells, crescents, fibrinoid necrosis, or endocapillary hypercellularity. The biopsy confirmed the diagnosis of amyloidosis without indications of SLE, Granulomatosis with Polyangiitis, or other disease processes associated with ds-DNA or PR3-ANCA. The patient was subsequently discharged after renal biopsy and continued treatment outpatient with bortezomib, cyclophosphamide, dexamethasone weekly, daratumumab every other week, and denosumab every three months.

## Discussion

AL amyloidosis is rare but is the most common form of amyloidosis. It is characterized by the extracellular deposition of beta-pleated sheets, i.e., amyloid light chain protein in various organs. Renal involvement is the most common and often presents with nephrotic syndrome, type II renal tubular acidosis, or nephrogenic diabetes insipidus. Diagnosis requires a renal biopsy to confirm the presence of amyloidosis-associated kidney disease. The underlying cause should be determined, and diagnostic workup may include serum protein electrophoresis, urine protein electrophoresis, urine-free kappa/lambda light chains, and bone marrow aspirate/biopsy. Serum protein electrophoresis was grossly normal in our patient, which is seen 25% of the time in individuals with AL amyloidosis. This is hypothesized to be due to the small quantities of light chains produced or the kidneys’ ability to filter out light chains entirely [5]. The patient's underlying etiology was confirmed with bone marrow biopsy, as ≥10% of the sample was comprised of plasma cells [6].

Anti-dsDNA antibody is highly specific for diagnosing systemic lupus erythematosus (SLE). Anti-dsDNA antibody has high specificity (97.4%) and poor sensitivity (57.3%) for SLE; however, these specificities and sensitivities carry different significance in the setting of renal disease: 41% and 65%, respectively [7]. However, there is a clear association between a high titer of immunoglobulin G anti-dsDNA antibodies and active lupus nephritis [8-10]. Anti-dsDNA antibodies are rarely found in patients with other disorders, including mixed connective tissue disease, uveitis, juvenile arthritis, antiphospholipid syndrome, Grave's disease, rheumatoid arthritis, Sjogren's syndrome, scleroderma, or Raynaud phenomenon [7,11]. 

There are two types of ANCA. The majority (90%) of ANCA are directed towards PR3. A minority (40%) of ANCA are against MPO-ANCA [12]. PR3-ANCA is most commonly found in those with Granulomatosis with Polyangiitis (80%) and less commonly in those with microscopic polyangiitis, Churg-Strauss syndrome, ulcerative colitis, and renal-limited rapidly progressive glomerulonephritis [13,14]. MPO-ANCA-associated crescentic glomerulonephritis has been shown in AL amyloidosis in a case study. Those with PR3-ANCA often have more upper-airway involvement and granulomatous lesions than MPO-ANCA, which exhibits more reno-pulmonary involvement [13].

A 2021 case did show MPO-ANCA positive crescentic glomerulonephritis with AL amyloidosis [4]. Similarities between the two patients include the advanced age of a Caucasian male, CKD, anemia, negative ANA titers, and cardiac involvement with left ventricular hypertrophy. Differences included our patient’s lack of crescentic involvement, our patient’s low light chain ratio (kappa/lambda ratio of 0.14 vs. 0.66), dsDNA negativity vs. positivity, the hypercellularity of our patient’s plasma cells (10%-20% vs. < 3%), and our patient’s low LVEF (47% vs. 55%-60%). As PR3-ANCA and ds-DNA involvement in AL amyloidosis has yet to be described in the literature, it is unclear if the presence of these antibodies affects the response to treatment or not. Additionally, these antibodies may or may not be associated with AL amyloidosis as they may have been related to the recent COVID-19 infection or be an early finding of a disease process yet to present clinically.

## Conclusions

Our case presents a unique and previously undocumented instance of AL amyloidosis in a patient with positive dsDNA and PR3-ANCA. The significance of these atypical antibodies remains unclear and could be associated with the underlying AL amyloidosis or an unknown drug exposure, recent infection, or another disease process. Further research is warranted to explore potential implications for diagnosis, prognosis, and treatment response. As clinicians understanding of rare conditions and their diverse manifestations continues to advance, cases like this one contribute to valuable insights for the medical community. Further studies and clinical observations may shed light on the significance of these antibody findings in AL amyloidosis and potentially inform future diagnostic and therapeutic approaches for similar cases.
